# A mixed-methods survey of physiotherapists who practice acupuncture and dry needling in Ontario, Canada: practice characteristics, motivations, and professional outcomes

**DOI:** 10.1186/s12906-021-03440-w

**Published:** 2021-10-19

**Authors:** Nadine Ijaz, Sandy Welsh, Heather Boon

**Affiliations:** 1 Department of Law and Legal Studies, Carleton University, 1125 Colonel By Drive, Ottawa, ON K1S 5B6 Canada; 2 Department of Sociology, University of Toronto, 725 Spadina Ave, Toronto, ON M5S 2J4 Canada; 3 Leslie Dan Faculty of Pharmacy, University of Toronto, 144 College Street, Toronto, Ontario M5S 3M2 Canada

**Keywords:** Physiotherapy, Physical therapy, Acupuncture, Dry needling, Professional education, Intramuscular stimulation, Motivations, Practice characteristics, Practice patterns, Training

## Abstract

**Background:**

Physiotherapists (PTs) across the globe are increasingly incorporating filiform needling techniques (e.g., acupuncture, dry needling) into their clinical toolkits; and, the evidence base for these complementary therapies is becoming progressively more robust. However, to date, little is known about needling PTs themselves.

**Methods:**

Using a cross-sectional survey design, PTs authorized to perform needling therapies in Ontario, Canada were recruited for anonymous participation (*n* = 2061) in an online survey. The survey asked providers about their demographics and practice characteristics, rationale for and views about therapeutic needling, and their related clinical and professional outcomes. The response rate was 20.7% (*n* = 426), and 22.3% (*n* = 95) of respondents provided textual responses to an open-ended qualitative question.

**Results:**

While study respondents’ demographic features appear similar to their broader professional population, Ontario’s needling PTs are less likely to work in public sector settings. Most completed training in medical acupuncture rather than dry needling, and typically used needling in over one-third of patient visits. Almost all endorsed needling as an effective musculoskeletal treatment, the primary factor informing their adoption of the practice. While many viewed traditional Chinese medical theories as a useful explanatory framework, most relied on biomedical epistemology to drive their needling work. A majority of respondents reported that the inclusion of needling within their clinical toolkits had improved their likelihood of achieving excellent clinical results, helped support patient recruitment and retention, and heightened their professional satisfaction. While a few reported earning a higher income as a result, most reported that their clinical use of needling in addition to other PT modalities reduced their physical fatigue after a day’s work.

**Conclusions:**

This study represents a first scholarly investigation into the motivations, training backgrounds and practice patterns of PTs who use acupuncture or dry needling. Additional research from other jurisdictions is needed to evaluate the transferability of study findings.

## Background

Filiform needling therapies such as acupuncture and dry needling are not generally included within core physiotherapy training curricula around the world, but physiotherapists’ (PT) use of these therapies to treat musculoskeletal disorders has been on the increase in recent decades. *Acupuncture* is a practice with historical roots in indigenous Chinese medicine that is today diversely practiced--including among PTs--both from within traditional East Asian [1] and biomedical [2] conceptual frameworks. Both historically and today, acupuncture has a range of therapeutic applications that include, but go well beyond, musculoskeletal pain. *Dry needling* broadly refers to a range of filiform needling techniques used primarily for treating musculoskeletal disorders from within a bioscientific epistemic framework. These techniques include ‘trigger point dry needling’, rooted in the saline injection therapeutics of American medical doctor Janet Travell [3], and ‘intramuscular stimulation’, an approach to pain treatment proposed by Canadian medical doctor Chan Gunn [4], informed by insights from traditional acupuncture. In this work, the term ‘needling’ or will be used to generically refer to all types of filiform needling practice, and more specific terms (e.g., dry needling) will be used to denote distinct needling approaches.

As early as 1984, needling was recognized as part of the PT profession’s statutory scope in New Zealand [5]; and in 1999, the World Confederation for Physical Therapy recognized the International Acupuncture Association of Physical Therapists as an official subgroup [6]. In 36 American states and across Australia, PTs are permitted to perform dry needling (but not acupuncture) with patients; and, PTs across Canada are variously permitted to use filiform needles within their statutory clinical scope [7]. Owing to the wide range of filiform needling approaches practised by PTs, and a lack of global agreement as to what constitutes appropriate related training requirements for PTs and other allied health professionals, standards of practice—where such exist—vary considerably across jurisdictions.

While it is generally agreed that the bodily insertion and manipulation of filiform needles by health professionals carries some associated risks, a growing body of scientific evidence related to the clinical effects of needling therapies continues to emerge [7]. Over 13,000 acupuncture-related clinical studies--one-third of which focused on pain treatment--were published between 1995 and 2014, at a publication rate twice that seen in conventional biomedical research [8]. Over two-dozen systematic reviews and meta-analyses of acupuncture’s effects on pain have been published by the Cochrane Collaborative alone, concluding overall that while more high-quality trials are needed, “acupuncture therapy is probably useful” [9]. More recently, trials focused on dry needling’s effects on musculoskeletal pain have emerged. Six related systematic reviews, published between 2005 and 2014 [10–15], generally reported heterogeneous results with respect to efficacy and characterized the quality of available evidence as low. At least 15 more such reviews and meta-analyses have been published since that time [16–29], over half of these in 2020 and 2021. These more recent analyses suggest low to moderate evidence for dry needling’s positive effects on various types of pain and compared with both placebo/sham and other active interventions. However, all reviews characterize the evidence as inconclusive, and call for additional dry needling research.

Although many clinical studies have investigated the effects of filiform needling as delivered specifically by PTs, just a few studies have examined the acupuncture-related characteristics and views of PTs themselves. Among Swedish PTs, one-third of surveyed PTs use acupuncture to treat low back pain and 44% use the practice to treat subacute neck pain [30]. Overall 89% viewed acupuncture as an effective pain treatment, with younger PTs significantly more likely to endorse the practice. A second Swedish study found that 56% of oncology-focused PTs treat patients using acupuncture (42% for cancer-related pain specifically) [31]. Fourteen percent of PTs studied in Saudi Arabia use acupuncture in clinical practice; and, usage was found to be more frequent among PTs with a stronger “biomedical” rather than “biopsychosocial treatment orientation” [32]. In the United Kingdom, 61% of surveyed PTs use acupuncture to treat contracted (frozen) shoulder [33]; and, at least a quarter use the technique to treat osteoarthritis of the hip [34]. Further, in a New Zealand pilot study, acupuncture was identified as PTs’ most widely-practised complementary medicine therapy [35]. The authors are unaware of any published workforce studies of PTs specifically identified to use dry needling rather than acupuncture in clinical practice. However, neither do the reviewed studies identify the specific filiform needling styles used by PTs; and, it is thus possible that existing PT workforce research may be conflating the range of needling approaches used within the profession.

To date, little is known about the practice characteristics of PTs who use therapeutic needling, their motivations for incorporating needling therapies into clinical practice, or their perception as to the impacts of this therapeutic approach on their clinical and professional outcomes. Addressing this gap, the current work presents a mixed-methods survey of physiotherapists who use therapeutic filiform needling in the province of Ontario, Canada. There, PTs who perform acupuncture or dry needling must be listed on their regulator’s public ‘roster’ for acupuncture [36]; however, the duration, type and content of needling-related training is left to the individual PT’s discretion. Furthermore, unlike some jurisdictions (such as across the United States), where PTs may only perform particular needling styles (e.g., ‘dry needling’) [37, 38], Ontario PTs have no such limitations.

## Methods

This work reports on a cross-sectional survey of PTs in Ontario, Canada who use filiform needles in clinical practice. Approval to conduct this study was granted by the University of Toronto’s Research Ethics Board. The funder, the Canadian Institutes of Health Research, played no role in the study’s design, conduct or reporting.

### Survey design

This survey formed part of a larger study of complementary medicine practitioners in Ontario, Canada, including four groups of acupuncture-practising professionals (PTs, massage therapists, naturopathic doctors and traditional Chinese medicine practitioners), as well as homeopathic practitioners. A number of questions were common across all of these surveys, including demographics, education and practice characteristics. Some survey items were adapted from those used by our team in a previous set of surveys of Ontario complementary medicine professions conducted in 2011–2012 [39]. Other items were informed by analyses of interviews with Ontario acupuncture practitioners and educators from across several professions, previously conducted by the first author [7, 40]. Survey questions were pre-tested with 14 registered members from across the aforementioned professions, including two licensed PTs. After review of pre-testing feedback, the research team incorporated appropriate changes to the surveys. Consistent with the terminology used by Ontario’s PT regulator [4] and the International Acupuncture Association of Physical Therapists [6], respondents were advised at the beginning of the survey that the term *acupuncture* would be being used to refer to all filiform needling approaches, including traditional and biomedical acupuncture, trigger point dry needling and intramuscular stimulation.

Constructed in adherence to the Tailored Design method [41], the authors constituted the present study survey as a predominantly-quantitative, mixed-methods design. The inclusion of a single (qualitative) open-ended question within our primarily-quantitative survey design had the primary aim of complementarity. As Greene and colleagues [42] explain, complementarity in mixed methods research “seeks elaboration, enhancement, illustration, clarification of the results of one method with the results from the other method”. In this process, researchers take advantage of the strengths of each of the employed methods; quantitative study findings are typically limited to the questions asked, whereas qualitative results can be more open-ended. In light of the limited prior research as to PTs’ motivations for, and clinical/professional experiences associated with practising needling therapies, the authors’ pursuit of complementarity not only aimed to provide nuance regarding the study’s quantitative results, but to provide additional analytic insights to inform future studies of this occupational group.

The survey included 52 quantitative questions interrogating the following points (a-c) and themes (d-f): a) demographics; b) needling-related training; c) needling-related practice characteristics; d) general views about needling; e) initial motivations for practising needling; f) self-reported clinical and professional outcomes related to needling practice. An open-ended question, posed at the end of the survey, invited respondents to provide additional comments about their use of needling.

### Sampling and recruitment

To achieve the best representation of this understudied population, the research team adopted a census- (rather than sampling-) style approach to recruitment. In early 2018, the authors used the provincial regulator’s online professional register [27] to construct a list of all PTs rostered to practice acupuncture in Ontario (*n* = 3137). By conducting public internet searches, the research team identified email addresses for 2129 providers. When the online survey was disseminated, 68 incorrect emails were returned, producing a total sample representing 65.7% (*n* = 2061) of Ontario’s needling PTs. In early 2019, we used the Qualtrics online platform to distribute the survey for anonymous, voluntary participation. All potential respondents were contacted by email, with reminders sent at one, three and 5 weeks after the initial recruitment notices were sent. The survey response rate was 20.7% (*n* = 426), with almost one-quarter of participants (22.3%, *n* = 95) providing textual responses to the open-ended question.

### Analysis

The authors undertook analysis of survey results with reference to a quantitative-dominant, crossover mixed analytic framework. Crossover mixed analytic designs are characterized by the analysis of more than one data type within a singular “paradigmatic tradition” [43]. In the present study, the authors—two PhD-credentialed health services researchers with a background in qualitative and mixed methods (NI, HB) and a quantitatively-focused, PhD-credentialed sociologist (SW)--adopted a postpositivist stance to analyse quantitative and qualitative data from the same survey.

The authors began the analytic process by conducting a descriptive statistical analysis of quantitative survey data, using Excel and SPSS statistical software. Participant responses to questions posed on a five-point Likert-scale were combined to report disagreement (“strongly disagree” and disagree”), neutrality (“neither agree nor disagree”) and agreement (“strongly agree” and “agree”). Not all respondents answered all survey questions; total n-values for all reported data are provided in the study tables.

Following statistical analysis, the first author (NI) used a thematic analytic approach, following Braun and Clarke [44], to code and categorize the full set of qualitative responses to the survey’s open-ended final question. NI’s scholarly background includes qualitative and mixed-methods studies of multiple occupations that use acupuncture in clinical practice, and formal training in critical qualitative health research methodologies— The initial deductive coding process began by identifying all open-ended survey responses that pertained to themes d), e), and f) identified under Survey Design above. At times, a single excerpt included content that pertained to multiple primary themes, and in such cases the excerpt was double-coded. Open-ended responses that did not directly pertain to themes d), e), and/or f) were separately grouped for subsequent analysis outside of the bounds of the present study, as elsewhere reported (cite, in press).

A secondary, more inductive analytic process of all thematically-coded excerpts followed. In this process, NI inductively categorized the deductively-coded excerpts from the study’s three primary thematic codes into sub-categories reflecting their specific content or focus. This secondary coding process aimed to comprehensively represent the full range of participant responses pertaining to the primary identified (deductive) themes. Coding was reviewed by other team members to address potential bias. Microsoft Excel and NVivo qualitative analysis software were used to manage the study’s qualitative dataset.

NI then identified representative textual excerpts for each of the study themes and sub-themes. In line with the study’s crossover design [43], qualitative findings—supported by representative textual excerpts—are presented alongside quantitative study results in what follows. Overall, these excerpts provide additional nuance to the analytic account, drawing attention to specificities not directly interrogated in the study’s quantitative questions and providing possible contextual explanations for some of the study’s numeric results.

## Results

### Demographics and practice characteristics

Table 1 details study participants’ demographic features and general practice characteristics. Participants’ mean age closely mirrors that of Ontario’s broader population of PTs (41.1 vs. 42.2 [45]). However, males are slightly over-represented (33.9% vs. 26.2% [46]) and professionals working part-time are under-represented in the study’s sample of acupuncture-providing PTs compared to the broader provincial PT population. Study participants had, on average, been using needling therapies in clinical practice for 10.7 years (SD 7.1), typically employed acupuncture needles in 38.4% of patient visits, treating 19.7 (SD 16.7) patients per week (Table 1). Whereas 40.9% of Ontario PTs work in hospital settings [46], just 5.3% of the needling PTs surveyed have their primary clinical practices in public sector locations.

**Table 1:** Demographics & Practice Characteristics of Physiotherapists Who Practice Needling

Factor	%	Mean (SD)
Gender (*n* = 371)		
*Male*^a^	33.9%	
Age (*n* = 364)		41.1 (9.8)
Annual income $CAD (*n* = 308)		$85,791($32,472)
Working < 36 h/wk. (*n* = 398)	11.6%	
Primary clinical site (*n* = 394)		
*Private clinic office*	88.8%	
*Public sector*	5.3%	
*Home office*	3.3%	
*Other (*e.g.*, home visits)*	2.5%	
Years using acupuncture / dry needling in clinical practice (*n* = 394)		10.7 (7.1)
% of client visits using needles (*n* = 398)	38.4%	
# of clients treated with needles / week (*n* = 394)		19.7 (16.7)

^a^Non-male respondents in the sample identified as either ‘female’ or ‘other’

### Needling-related training backgrounds

Data pertaining to participants’ needling-related trainings are shown in Table 2. The majority of participants (62.8%) received 1 hundred or fewer hours of needling-related training. The majority of training programs emphasized a ‘medical acupuncture’ (72.4%) rather than a ‘dry needling’ or ‘intramuscular stimulation approach’; further, over 100 respondents had completed more than one, and/or more than one type of training program. While most participants (90.1%) expressed satisfaction with their acupuncture-related knowledge, three-quarters also expressed interest in further education related to biomedical needling approaches including dry needling and intramuscular stimulation. Although three-quarters of participants’ trainings had included a component of traditional Chinese medical theory, just 30.0% expressed interest in further education in this area.

**Table 2:** Needling-Related Training Completed by Physiotherapists who Practice Needling

Factors	%
Duration of formal needling training (*n* = 392)	
*Fewer than 100 h*	62.8%
*101 + hours*	37.2%
Type of training (*n* = 517)^a^	
*Medical acupuncture*	59.8%
*Dry needling / intramuscular stimulation*	22.8%
*Other / unspecified*	17.4%
Training included traditional Chinese medical theory (*n* = 393)	76.1%
Interest in further education	
*Satisfied with current needling knowledge* (*n* = 384)	90.1%
*Interested in furthering education in contemporary biomedical needling approaches (*e.g.*, medical acupuncture, dry needling, intramuscular stimulation)* (*n* = 384)	75.2%
*Interested in furthering education in traditional Chinese medicine* (*n* = 381)	30.0%

^a^Number of trainings exceeds number of participants as some participants completed multiple trainings

### General views about needling as a musculoskeletal treatment

Overall, as seen on Table 3, virtually all surveyed respondents (over 95%) endorsed needling as an effective and safe treatment for musculoskeletal disorders, and believed the practice belongs within the PT profession’s scope of practice. However, a small number disagreed or expressed neutrality on these issues. Qualitative survey responses provide insight into the possible basis for such non-endorsement, indicating that some PTs perceive the available scientific evidence to poorly substantiates the use of needling therapies:

*Currently the body of research doesn’t show any greater improvements with the use of acupuncture vs. sham, so I have substantially cut down my use as it's not supported.*

*Acupuncture is overused by too many physiotherapists, and physiotherapists should not be allowed to perform Chinese medicine-based acupuncture. This type of acupuncture has no scientific basis, and even the western biomedical approach has a shaky evidence base.*

While three-quarters of participants agreed that acupuncture could be effectively explained using biomedical scientific perspectives, just over half viewed traditional Chinese medical perspectives about acupuncture as clinically useful. In their open-ended responses, a number of respondents emphasized that acupuncture and dry needling represent significantly different approaches to treatment, both in theory and practice:

*Dry Needling and Acupuncture [are] different practices with different underlying models of clinical efficacy. They are similar only in that they both use a needle.*

*I am sure to explain to all my clients that I am performing a technique that I am trained and qualified in called Dry Needling which uses the same needles as acupuncture needles, but that I am not doing TCM [traditional Chinese medical] acupuncture.*

Echoing survey findings, some respondents furthermore expressed personal views about the comparative effectiveness of one versus the other -- or argued that both had potential to exert good clinical effect.

*Dry needling is more effective than standard acupuncture and I am getting far better results with that with my patients.*

*I think that a combined approach of acupuncture and dry needling is effective…*[It] *is helpful to have the option of acupuncture during more acute phases where manual therapy or needling would be too aggressive.*

**Table 3:** General Perspectives of Physiotherapists Who Practice Needling

Factors	Agree	Neutral %	Disagree
*Needling is an effective treatment for musculoskeletal disorders* (*n* = 385)	93.8%	3.9%	2.4%
*Needling is a safe treatment for musculoskeletal disorders* (*n* = 385)	97.7%	1.0%	1.3%
*Needling belongs in the scope of practice of PTs* (*n* = 385)	95.1%	2.1%	2.6%
*Traditional Chinese medical perspectives about needling are clinically useful* (*n* = 385)	57.5%	23.7%	18.8%
*Needling’s effects can be effectively explained using modern Western medical perspectives* (*n* = 385)	75.1%	9.6%	15.4%

### Motivations for incorporating needling into clinical practice

As shown in Table 4, the vast majority of respondents indicated that they had initially incorporated needling into their clinical toolkits based on a belief in the technique’s effectiveness in treating for musculoskeletal conditions. About half hoped it would help them attract more patients and one-third hoped it would provide them, as clinicians, some relief from their other, more physically-demanding therapeutic modalities. In the survey’s open-ended responses, several respondents additionally cited patient demand as a specific motivation for their professional use of needling:

*Clients are seeking acupuncture for pain reduction because they are having little success with other methods or for medical reasons are not able to use the majority of pain medications.*

*Patients call and request acupuncture because they heard it worked for someone else.*

**Table 4:** Motivations for Using Needling in Physiotherapy Practice

Factors	Agree	Neutral %	Disagree
*I thought it would be an effective treatment for musculoskeletal conditions* (*n* = 377)	97.6%	1.6%	0.8%
*I hoped it would help me attract more patients* (*n* = 370)	49.5%	30.0%	20.6%
*I was finding it physically tiring to apply my other therapeutic techniques* (*n* = 371)	32.1%	24.3%	43.7%
*I was trained in needling before I beganworking as a PT* (*n* = 360)	7.2%	8.3%	84.5%
*Other* (*n* = 46)	52.2%	21.7%	26.1%

### Self-reported clinical outcomes

Table 5 elaborates upon the professional outcomes reported by respondents in relation to their clinical use of needling. Overall, just under three-quarters indicated they were more likely to achieve excellent clinical results, a point re-iterated across the survey’s qualitative responses:

*Acupuncture is one of the best pain modalities used in my physiotherapy practice, which allows me to obtain excellent clinical results and return my patients to their previous functional levels.*

*IMS [intramuscular stimulation] or dry needling is probably the number 1 clinical tool for me.*

Some attributed their increased clinical successes to the combination of needling with other PT techniques:

*Dry needling combined with manual therapy and corrective exercises has changed my practice tremendously! In my opinion it is the most effective way to treat musculoskeletal / movement disorders / injuries.*

Across the qualitative dataset, participants made clear that they used needling to successfully treat a wide range of clinical issues including musculoskeletal “pain relief”, motor vehicle accident care, and nervous system ailments. For example:

*It's great for muscle re-activation, decreasing pain and swelling, and it quickens recovery time. You can get very fast results with muscular injuries especially when using electro-acupuncture.*

*[Acupuncture] works very well with MVA [motor vehicle accident] clients and similar who can often not tolerate more intensive treatment methods. It also provides good reductions in pain and improvements and mobility.*

*Often I turn to acupuncture for balancing the nervous system in conditions like whiplash. I have found it invaluable in this population and nerve related issues.*

There were no qualitative responses in which the surveyed PTs detailed poor clinical outcomes from the use of needling. Notably, 41.6% of respondents indicated they were more likely to have adverse events in clinical practice since incorporating needling into their clinical toolkits but did not elaborate further on this point in their qualitative responses.

**Table 5:** Self-Reported Clinical & Professional Outcomes of Physiotherapists who Practice Needling

Factors	Agree	Neutral %	Disagree
**Since incorporating needling into my clinical practice, I am:**			
***Clinical Outcomes***			
*More likely to achieve excellent clinical results* (*n* = 377)	72.5%	12.2%	5.3%
*More likely to achieve excellent clinical results* (*n* = 377)	72.5%	12.2%	5.3%
*More likely to have adverse events in my clinical practice* (*n* = 375)	41.6%	51.2%	7.2%
***Professional Outcomes***			
*More satisfied with my professional work* (*n* = 376)	75.8%	19.7%	4.5%
*Earning a higher income* (*n* = 377)	22.3%	35.3%	42.4%
*More likely to attract new patients* (*n* = 377)	67.4%	24.1%	9.5%
*More likely to retain existing patients* (*n* = 376)	68.6%	22.9%	8.5%
*Less physically tired after a day’s work* (*n* = 373)	75.1%	23.3%	1.6%

### Self-reported professional outcomes

As further shown on Table 5, three-quarters of respondents indicated that they were more satisfied with their professional work since incorporating needling into their clinical toolkits. While just one-fifth reported earning a higher income as a result, about two-thirds indicated they were more likely to attract and retain patients with needling as one of their therapeutic modalities. We identified no qualitative responses associated with the aforementioned points.

Although just one-third of participants had initially been motivated to practice needling because they were finding their other therapeutic techniques physically tiring (Table 3), three-quarters noted that the inclusion of needling in their clinical toolkits resulted in them being less physically tired after a day’s work (Table 5). A few qualitative responses echoed these findings:

*I love having acupuncture in my toolkit. It sometimes takes the place of muscular release techniques, which helps save my hands.*

*It is extremely helpful in getting patients better quicker and with less pain. It also allows for physiotherapist to take less stress on our body.*

## Discussion

This study represents a first scholarly investigation into the practice characteristics, motivations, and self-reported clinical and professional outcomes of PTs who use acupuncture or dry needling as an adjunct modality in clinical practice. Whereas prior workforce studies addressing PTs’ use of needling therapies have surveyed members of the profession more broadly, this study uniquely recruited PTs self-identified to be needling practitioners. Demographically, needling PTs are similar to others within the profession, but differ in that they use needling, on average, in over one-third of patient visits. The study finding that members of this occupational subset are considerably less likely to work in public sector settings is a point warranting further investigation; but, might conceivably relate to a need for stronger scientific evidence for this complementary medicine practice to be endorsed or accepted in state-funded clinical environments.

Study findings point to a primary epistemic endorsement of bioscientific perspectives on needling among surveyed PTs, despite three-quarters of respondents having received training that included a component of traditional Chinese medicine theory. On one hand, that over half of respondents viewed Chinese medical perspectives about needling as clinically useful suggests a considerable respect for traditional acupuncture’s non-biomedical epistemology among Ontario’s needling PTs. Nevertheless, just a minority expressed interest in additional Chinese medicine training, as compared to a strong majority that not only endorsed bioscientific explanatory frameworks for needling but also showed enthusiasm for further biomedically-focused needling education.

Study respondents’ almost-universal endorsement of needling as an effective treatment for musculoskeletal disorders contrasts with lower rates of needling endorsement previously reported within the larger PT profession [30, 32, 35]. This disproportionately high enthusiasm for the practice is likely a reflection of the occupational subset of PTs surveyed, all of whom had elected to complete training in needling themselves. Qualitative responses from among the small minority of study respondents who, despite their needling training, did not see the practice as an effective treatment suggest that the inconclusive evidence base for needling as a musculoskeletal pain treatment may be of concern among some PTs, even as this evidentiary base continues to grow.

That almost three-quarters of study respondents reported that they were more likely to achieve excellent clinical results with needling in their treatment toolkits aligns with the growing body of scientific evidence substantiating both acupuncture and dry needling as an effective pain treatment. Further aligned with the evidence base are respondents’ anecdotal reports of needling’s utility in treating a range of musculoskeletal conditions. Nevertheless, that over 40 % of respondents reported heightened incidence of adverse effects in clinical practice since starting to use needling is a finding that may warrant additional research – and perhaps, as elsewhere recommended [7], a heightened focus on safety in regulators’ needling-related training and practice standards.

Respondents generally affirmed that their use of therapeutic needling as a clinical tool had advantageous outcomes beyond the clinical, supporting them as practitioners to not only recruit and retain patients, but also to enhance their overall professional satisfaction. Echoing the result of a recent survey involving Ontario’s acupuncture-practising massage therapists [47], this enhanced satisfaction was notably *not* accompanied by an increase in income but rather, in most cases, by a reduction in overall physical fatigue after a day’s work. That the inclusion of filiform needling therapies in Ontario allied health professionals’ treatment toolkits appears, by self-report, to enhance clinical outcomes and professional satisfaction but not improve clinicians’ income-generating power is a notable finding warranting additional exploration.

A strength of this survey-based study comes from the high level of responses to the qualitative, open-ended question posed of participants. The range of themes and sub-themes elucidated across these responses permitted a more nuanced interpretation of quantitative findings than may have otherwise been possible. Qualitative responses furthermore suggest areas of intra-professional disagreement that call for further investigation moving forward.

The study also has limitations. Recall bias may be a factor, given that all data are self-reported. In addition, the transferability of findings to other jurisdictions may be limited. In particular, in the United States, where PTs are generally prohibited from practising ‘acupuncture’, a larger proportion of providers’ trainings would likely have been focused in the area of dry needling or intramuscular stimulation, rather than medical acupuncture. Why Ontario’s needling PTs would have predominantly completed trainings in medical acupuncture may be an artefact of the locally-available trainings, a reflection of provider interest, or some other as-yet unexplored factor.

## Conclusion

This study represents a first scholarly investigation into the motivations, training backgrounds and practice patterns of PTs who use acupuncture or dry needling. Overall, respondents used needling with about one-third of their patients, typically in private sector settings. Most endorsed the practice as both clinically effective and professionally advantageous. Additional research from other jurisdictions is needed to evaluate the transferability of study findings about this important sub-trend within the PT profession. Future research should attend more directly to potential differences between practitioners of diverse needling approaches.

## Data Availability

The datasets used and/or analysed during the current study are available from the corresponding author on reasonable request, per the stipulations of the University of Toronto Research Ethics Board approval.

## Abbreviation

PT: Physical Therapist
